# Introduction to neonatologist-performed echocardiography

**DOI:** 10.1038/s41390-018-0076-y

**Published:** 2018-08-02

**Authors:** Alan M. Groves, Yogen Singh, Eugene Dempsey, Zoltan Molnar, Topun Austin, Afif El-Khuffash, Willem P. de Boode, on behalf of the European Special Interest Group ‘Neonatologist Performed Echocardiography’ (NPE), K Bohlin, M C Bravo, C R Breatnach, M Breindahl, S Gupta, B Horsberg Eriksen, P T Levy, P J McNamara, E Nestaas, S R Rogerson, C C Roehr, M Savoia, U Schubert, C E Schwarz, A Sehgal, Y Singh, M G Slieker, C Tissot, R van der Lee, D van Laere, B van Overmeire, L van Wyk

**Affiliations:** 1 Division of Newborn Medicine, Mount Sinai Kravis Children’s Hospital, New York, NY USA; 2 Addenbrooke’s Hospital, Cambridge University Hospitals NHS Foundation Trust, Cambridge, United Kingdom; 3 INFANT Centre, Cork University Maternity Hospital, University College Cork, Cork, Ireland; 4 John Radcliffe Hospital, Oxford, United Kingdom; 5 Department of Neonatology, Rosie Hospital, Cambridge University Hospitals NHS Foundation Trust, Cambridge, United Kingdom; 6 Department of Neonatology, The Rotunda Hospital, Dublin, Ireland; 7 Department of Pediatrics, The Royal College of Surgeons in Ireland, Dublin, Ireland; 8 Department of Neonatology, Radboud University Medical Center, Radboud Institute for Health Sciences, Amalia Children’s Hospital, Nijmegen, The Netherlands; 9 Department of Neonatology, Karolinska University Hospital, Karolinska Institutet, Stockholm, Sweden; 10 Department of Neonatology, La Paz University Hospital, Madrid, Spain; 11 Karolinska University Hospital, Karolinska Institutet, Stockholm, Sweden; 12 University Hospital of North Tees, Durham University, Stockton-on-Tees, United Kingdom; 13 Department of Pediatrics, Møre and Romsdal Hospital Trust, Ålesund, Norway; 14 Department of Pediatrics, Washington University School of Medicine, Saint Louis, MO USA; 15 Department of Pediatrics, Goryeb Children’s Hospital, Morristown, NJ USA; 16 Departments of Pediatrics and Physiology, University of Toronto, Toronto, ON Canada; 17 Institute of Clinical Medicine, Faculty of Medicine, University of Oslo, Oslo, Norway; 18 Department of Cardiology and Center for Cardiological Innovation, Oslo University Hospital, Rikshospitalet, Oslo, Norway; 19 Department of Paediatrics, Vestfold Hospital Trust, Tønsberg, Norway; 20 The Royal Women’s Hospital, Parkville, VIC Australia; 21 Department of Paediatrics, University of Oxford, John Radcliffe Hospital, Oxford, United Kingdom; 22 Azienda Ospedaliero-Universitaria S. Maria della Misericordia, Udine, Italy; 23 Department of Clinical Science, Intervention and Technology, Karolinska Institutet, Stockholm, Sweden; 24 Department of Neonatology, University Children’s Hospital of Tübingen, Tübingen, Germany; 25 Department of Pediatrics, Monash University, Melbourne, Australia; 26 Department of Paediatric Cardiology, Radboudumc Amalia Children’s Hospital, Nijmegen, The Netherlands; 27 Department of Pediatrics, Clinique des Grangettes, Chêne Bougeries, Switzerland; 28 Department of Pediatrics, Antwerp University Hospital UZA, Edegem, Belgium; 29 Department of Neonatology, University Hospital Brussels, Brussels, Belgium; 30 Department of Paediatrics & Child Health, University of Stellenbosch, Cape Town, South Africa

## Abstract

Cardiac ultrasound techniques are increasingly used in the neonatal intensive care unit to guide cardiorespiratory care of the sick newborn. This is the first in a series of eight review articles discussing the current status of “neonatologist-performed echocardiography” (NPE). The aim of this introductory review is to discuss four key elements of NPE. Indications for scanning are summarized to give the neonatologist with echocardiography skills a clear scope of practice. The fundamental physics of ultrasound are explained to allow for image optimization and avoid erroneous conclusions from artifacts. To ensure patient safety during echocardiography recommendations are given to prevent cardiorespiratory instability, hypothermia, infection, and skin lesions. A structured approach to echocardiography, with the same standard views acquired in the same sequence at each scan, is suggested in order to ensure that the neonatologist confirms normal structural anatomy or acquires the necessary images for a pediatric cardiologist to do so when reviewing the scan.

## Introduction

Clinicians have been utilizing echocardiographic techniques in the neonatal intensive care unit (NICU) since the 1990s^1^ and there has been a significant increase in the application of this technology in the last decade.^2,3^ The goal of “neonatologist-performed echocardiography” (NPE) is to use ultrasound to guide improved cardiorespiratory care of the sick newborn.^4^

The aim of this review article is to discuss four key elements of NPE:

1. *Indications for scanning*: These have been suggested in a number of different consensus statements,^4–6^ and will be reviewed here to provide an introduction to the judicious application of NPE.

2. *Physics of ultrasound*: An awareness of ultrasound physics is essential to understand the limitations of the technique and to avoid being misled by common image artifacts.

3. *Patient safety during echocardiography*: An area that, particularly when considering cardiorespiratory stability and infection control, cannot be taken lightly.

4. *Routine echocardiographic views*: These views form the basis for both confirming normal structural anatomy and assessing hemodynamic status.

## Indications for NPE

The principal indications for NPE are discussed below. Irrespective of the indications we support the views of the American Society of Echocardiography (ASE), European Association of Echocardiography (EAE), the Association for European Paediatric Cardiologists (AEPC);^4^ the British Congenital Cardiac Association (BCCA);^5^ and the European Society for Pediatric Research (ESPR)/European Society for Neonatology (ESN)^6^ that the first echocardiogram performed in a newborn should include a comprehensive structural assessment to confirm normal structural anatomy, even when the suspicion of structural lesions is low. Any child in whom structural congenital heart disease is suspected should have appropriate referrals made and appropriate therapy initiated, as would be the case based on clinical suspicion alone.

As the level of experience of the neonatologist performing the scan, and the availability of a pediatric cardiologist will be variable, different centers will adopt different approaches to confirm normal structural anatomy. In this review we focus on the acquisition of routine standard echocardiographic views to support an approach for the clinicians performing NPE to confirm normal structural anatomy, with timely review of the study by a pediatric cardiologist. Having accepted the need for confirmation of normal structural anatomy with the first assessments, the most common indications for NPE are discussed below.

### Patent ductus arteriosus

There is general consensus that treatment should be based on an estimate of the hemodynamic significance of the patent ductus.^7^ A comprehensive echocardiographic assessment, including the exclusion of ductal-dependent structural lesions, is increasingly accepted as best practice.^8^ Serial echo assessments can also guide duration of therapy and lead to a reduction in medication doses (Bravo,^9^ see van Laere et al., this issue).

### Persistent pulmonary hypertension of the newborn

Differentiating persistent pulmonary hypertension (PPHN) from cyanotic congenital heart disease is clinically challenging, and an early echocardiogram to confirm the diagnosis allows the clinical team to focus entirely on optimal PPHN management without retaining doubts over a structural lesion.^10^ NPE can provide invaluable insights into heart function, cardiac output, and cardiac loading conditions;^10^ repeat scans may offer insights regarding treatment response (see de Boode et al., this issue).

### Neonatal shock/hypotension

Inadequate end-organ perfusion may occur both in the immediate postnatal period^11^ and also later during the neonatal course as a result of sepsis/necrotizing enterocolitis,^12,13^ or patent ductus arteriosus (PDA) ligation.^14^ NPE assessments of cardiac filling, function, output, and systemic perfusion can guide decision making for fluid resuscitation and initiation of inotropes and/or vasopressors (Mertens et al.,^4^ see de Boode et al., this issue).

### Feto-neonatal transition

NPE can be used during the feto-neonatal transition in the first 12 to 24 h after birth in preterm infants <28 weeks’ gestation for the assessment of systemic blood flow and ductal status, since during this period these babies are hemodynamically very vulnerable, often without overt clinical signs.

### Line placement

NPE may have a role in confirming correct positioning of both umbilical and peripherally inserted central lines in the newborn.^4^ However, it is noted that these techniques show high user dependency, with identification of the true tip of a line often challenging. Some centers also opt to use NPE to identify line-associated thrombus.

### Identification of pericardial effusion

This rare, but potentially catastrophic, complication is often associated with intracardiac placement of an indwelling line. Rapid identification of pericardial effusion in an acutely unwell newborn can allow therapeutic pericardiocentesis, which again can be guided by ultrasound.^4^

## The physics of ultrasound

In our experience novice scanners are often intimidated by the need to understand the principles of physics when learning ultrasound. However, it is vital that the user of the ultrasound machine understands the basics of how images are obtained and reconstructed so as to appreciate the limitations of the technology and understand common artifacts. In this section, we provide an overview of imaging physics and refer the reader to more comprehensive texts for additional detail.^15–18^

### Characteristics of ultrasound waves

**Fig. 1:**
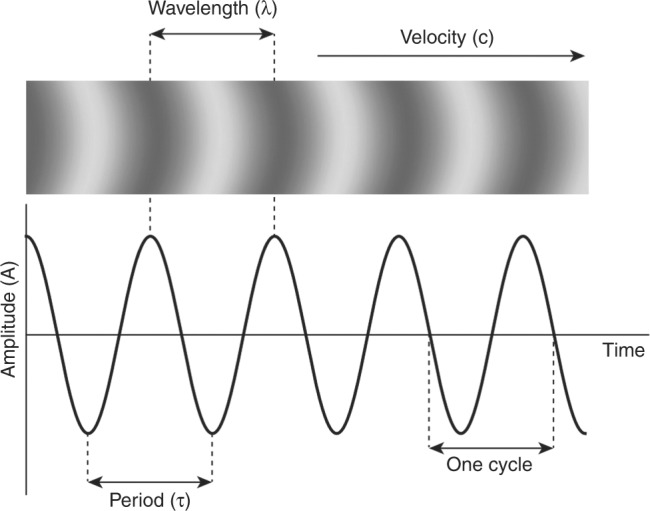
Parameters of a sound wave

Sound waves are described in terms of their wavelength (*λ*), the distance between two points of the same phase; frequency (*f*), the number of cycles per second, measured in hertz (Hz); amplitude (*A*), maximum particle displacement, measured in decibels (dB); and velocity (*c*), the speed of propagation through a given medium, measured in m/s (Fig. 1). Velocity in turn depends on the physical characteristics of the medium and can be calculated by applying the following formula:

$$c = \sqrt {\frac{\beta }{\rho }},$$

where *β* denotes stiffness and *ρ* denotes *density*. The average speed of ultrasound in soft tissues is around 1500 m/s, with slight differences between tissue types. The wave equation dictates that velocity of sound is proportional to the wavelength (*λ*) and the frequency (f) ($c \propto f \times \lambda$). Given that the velocity of ultrasound in soft tissues is relatively constant then frequency can be assumed to be inversely related to wavelength ($f \propto c/\lambda$).

### Generation of ultrasound

In the imaging setting, ultrasound waves are generated by transducers equipped with piezoelectric crystals. These crystals change shape when electric currents are applied through them, and similarly, they generate electric signals upon mechanical compression. Individual crystals are located adjacent to each other in an “array” and are connected electrically.

**Fig. 2:**
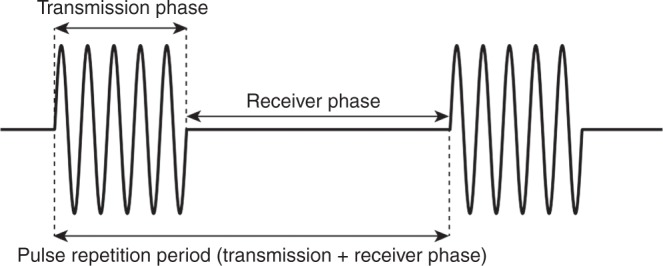
Pulse duration and pulse repetition period. Adapted from Rovner A. The principle of ultrasound—ECHOpedia 2017. Available from: http://www.echopedia.org/wiki/The_principle_of_ultrasound

Applying rapid alternating current to the crystals generates vibration and ultrasound emission. This “transmission phase” is very brief (0.5–3 μs) and is followed by a “receiver phase” in which returning sound waves compress the piezoelectric crystals and generate electric signals. This phase is much longer (up to 1 ms) than the transmission phase since echoes from a range of depths must be detected. The combined durations of the transmission and receiver phases is the pulse repetition period; a shallower depth allows for a shortened receiver phase and therefore a shorter pulse repetition period and a higher frame rate (Fig. 2).

### Types of probes

Ultrasound probes are available in a variety of types of array. In echocardiography, a phased array transducer is generally used because of its small footprint, allowing imaging through small intercostal windows. Phased array probes can be steered and focused to further optimize imaging. Some vascular applications favor a linear array for maximal spatial resolution.

### Interaction of ultrasound with tissues

**Fig. 3:**
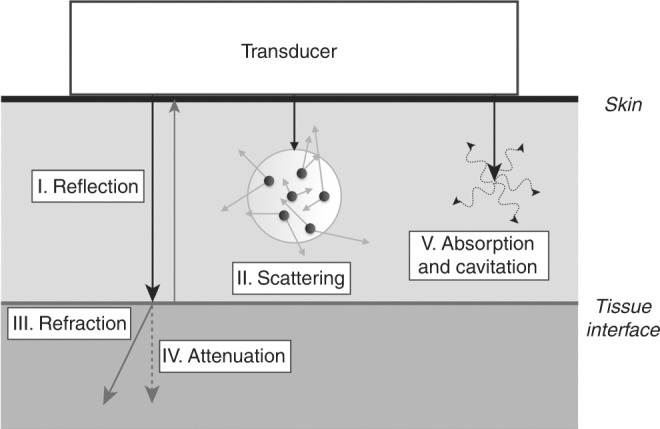
Ultrasound and tissue interactions. Adapted from Textbook of Clinical Echocardiography, 5th Edition, Catherine Otto, “Principles of Echocardiographic Image Acquisition and Doppler Analysis,” Fig. 1.4, page 5, Copyright (2013), with permission from Elsevier

Interactions between emitted ultrasound waves and tissues are what produce the images. These interactions may be of different types, an awareness of which is key to understanding common image artifacts (Fig. 3).

1. *Reflection*: When an ultrasound beam hits a boundary/interface between two different tissues, part of the ultrasound is reflected back to the probe. The amount of reflection depends on the difference in the acoustic properties of the two tissues, specifically the acoustic impedance, which is mainly a product of tissue density. It is the significant difference between density of soft tissue and air that prevents ultrasound being able to image through overlying lung or pneumothorax. The magnitude of returning reflection is also influenced by the angle between the tissue border and the ultrasound beam. Maximal reflection is obtained when tissue border is orthogonal to the ultrasound beam. A clear demonstration of this property is in imaging the membranous intraventricular septum from the four-chamber view (see below) when the false impression of a septal defect can be made since there is so little reflection from a structure that is almost in line with the ultrasound beam. Hence, the ventricular septum should be interrogated from a subcostal or parasternal view, where the septum is orthogonal to the beam.

2. *Scattering*: When an ultrasound beam meets a boundary consisting of small structures (smaller than the wavelength of the sound), the ultrasound beam is scattered. This results in reflection of the beam to all directions and a disorganized returning signal. Most of the signal is lost due to the scattering in multiple directions. Nonetheless, backscattering plays an important role in generating the eventual two-dimensional (2D) image and most organs have a characteristic scatter signature owing to their specific structures. Hyperechoic (bright) regions within an organ usually represent increased scattering.

3. *Refraction*: Refraction refers to the bending of the ultrasound beam when it enters a medium where its propagation speed is different (as is seen when looking at an object below the surface of water). The degree of bending depends on the angle between the beam and the surface (angle of insonation), and the degree of difference in propagation speeds between tissues. Refraction artifact may cause objects to appear in altered locations.

4. *Attenuation*: As ultrasound travels within tissues, part of the energy is lost to absorption and scattering. This results in weaker signal intensity from structures that are farther from the probe. The higher the frequency, the greater the attenuation, and therefore the lower the penetration. Modern scanners use automatic “time gain compensation” to ameliorate this problem.

5. *Absorption and cavitation*: Absorption of ultrasound by human tissues is the process of energy loss by conversion to heat. Cavitation occurs when microbubbles are formed due to high-energy ultrasound interaction. All clinical ultrasound systems work within carefully controlled energy settings, such as those set by the US Food and Drugs Administration,^19^ and ultrasound imaging is not considered to have any biologic ill effects. Since all imaging introduces energy into the body, imaging power and duration of scans should be kept to a minimum.

### Image production

Returning ultrasound waves lead to compression of the piezoelectric crystals and are converted to an electric signal and processed to produce an image on the screen. In B mode (Brightness mode) a 2D image is produced which is a representation of an anatomic slice of tissue. In M mode (Motion mode), one dedicated scan line is used to detect rapidly moving structures. This form of imaging provides the highest temporal and spatial resolution.

Harmonic imaging is applied in echocardiography to resolve the potential influence of tissue resonance on image quality. An ultrasound wave that penetrates the body will lead to resonance of tissue. The frequency of this resonance is characteristically a multiple of the initial transmitted frequency. Since these harmonic frequencies are also being reflected in tissue, they contribute to the creation of the two-dimensional picture. In harmonic imaging, all harmonic frequencies are filtered, except for the second harmonic component of the original signal, resulting in a higher resolution and fewer artifacts. Harmonic imaging is widely used in adult echocardiography, as it results in better signal-to-noise ratio. As it has poorer axial resolution, it is not widely applied in newborns.

### Resolution

**Fig. 4:**
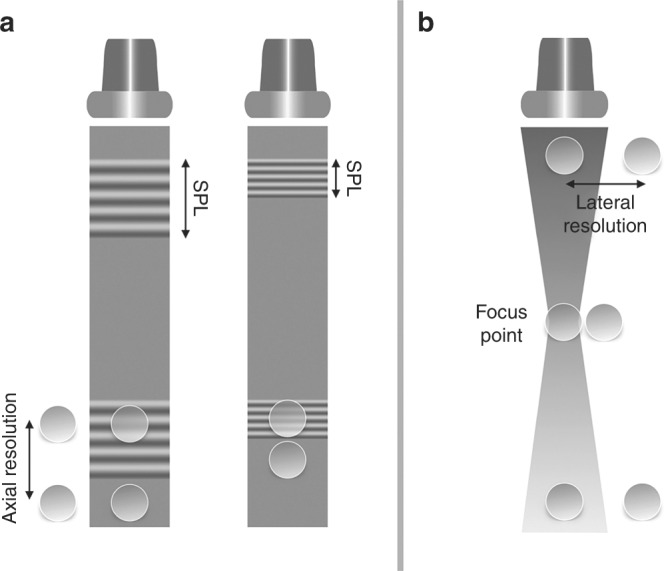
Axial (left panel) and lateral (right panel) resolution. See text for details. Adapted from Rovner A. The principle of ultrasound—ECHOpedia 2017. Available from: http://www.echopedia.org/wiki/The_principle_of_ultrasound

Resolution of ultrasound imaging includes both spatial and temporal resolution. Spatial resolution is further divided into axial, lateral, and elevational resolution. Axial resolution is the ability to differentiate structures that are aligned along the imaging beam (Fig. 4). Axial resolution is determined by spatial pulse length (SPL), which is the product of wavelength and the number of cycles in one pulse. The lower the SPL, the higher the resolution. Increasing the frequency decreases the wavelength, therefore yielding better resolution. Typical axial resolution is 0.5 mm at a transmitting frequency of 5 MHz and 0.25 mm at 10 MHz.

Lateral resolution is the ability to discriminate objects located in an axis perpendicular to the ultrasound beam (Fig. 4). The major determinant of lateral resolution is beam width. Focusing the transmitted beam by applying the electric current to the individual piezoelectric crystals with time delay decreases the width of the beam at the focal point, thereby improving lateral resolution. The focus position can be set by the operator and is one of the key steps in image optimization. Multiple focal points yield more homogenously distributed lateral resolution in the 2D image, but comes at the expense of a decrease in frame rate. Lateral resolution is best at shallow depths and narrow beams and worse with deeper imaging and wide beams.

Temporal resolution is the ability to detect that an object has moved over time; it is described in terms of frame rate, in Hz or frames/s. Frame rate depends on the time taken to create a single image line, and the number of lines that form each image. Frame rate can therefore be improved by decreasing the imaging depth, narrowing the image sector width, zooming into an area of interest, reducing the number of focus points, or decreasing the line density of the sector.

### Artifacts

In pursuit of an accurate representation of anatomy, the ultrasound machine makes a number of assumptions about sound propagation in tissue. Artifacts are errors in image production and are normally caused by physical processes that affect the ultrasound beam. Recognizing imaging artifacts is of great importance to prevent misinterpretation of echocardiograms. A key principle of all imaging is a constant awareness of the possibility of image artifacts. Artifacts can often be recognized by altering the image plane, depth, or frequency. Any unusual object should be viewed from multiple directions to ensure that it is anatomic rather than artifactual.

**Fig. 5:**
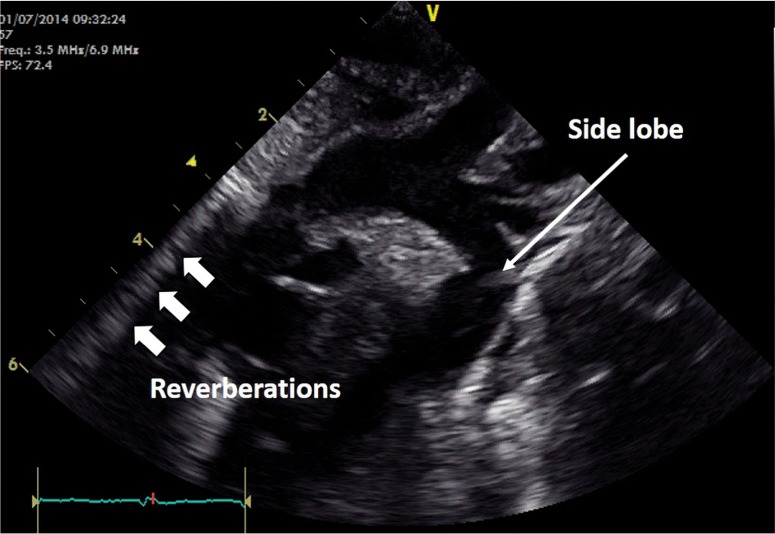
Arch view in a newborn showing reverberation and sidelobe artifacts

**Fig. 6:**
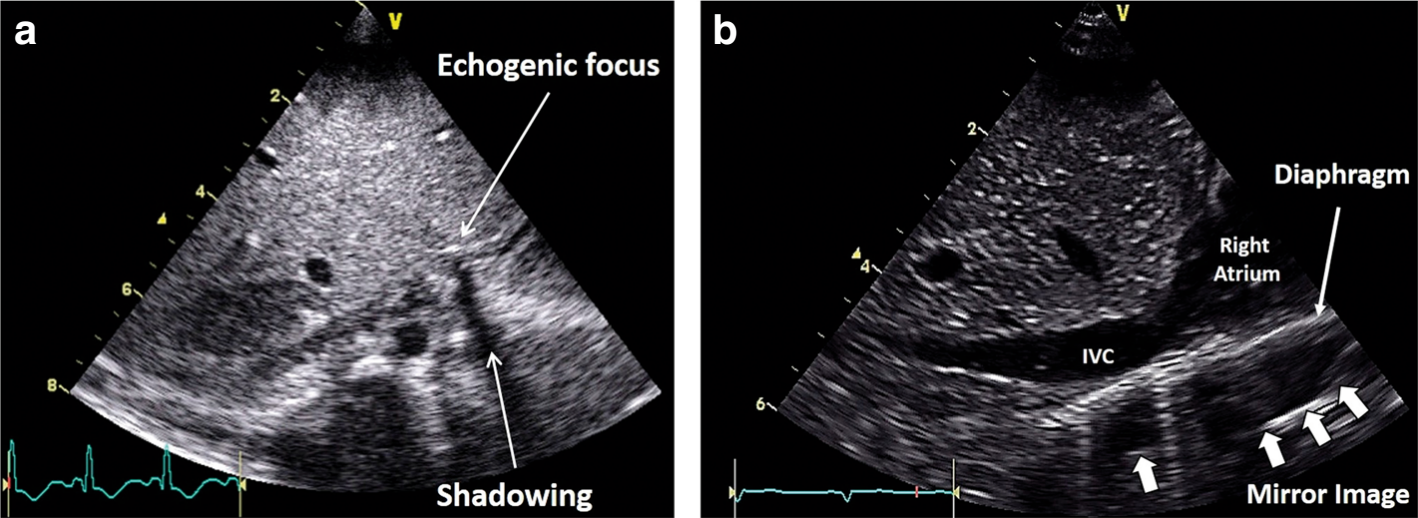
A subcostal situs view demonstrating an echogenic focus in the liver and shadow artifact (**a**) and a subcostal parasagittal view of the liver and inferior vena cava (IVC) demonstrating a mirror image against the diaphragm (**b**)

**Fig. 7:**
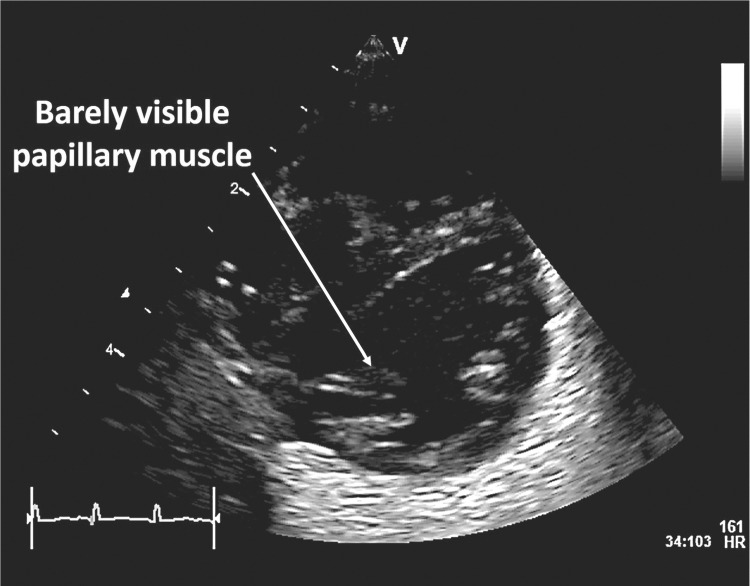
An example of beam width artifact in the parasternal short axis view at the level of the papillary muscle

1. Reverberation artifacts are generated by strong reflectors, such as the ribs or pericardium, when waves do not travel directly to and from a tissue but have additional reflections within the tissue (Fig. 5) before returning to the echo probe. Since the echo transducer assumes that waves have taken a direct path to the tissue and back reverberation artifacts appear as multiple images behind reflectors or “comet tails.”

2. Side lobe artifacts—scanners display a 2D representation of tissue on the ultrasound screen assuming the ultrasound beam is infinitely thin. However, this is not the case and objects in front or behind the 2D plane being imaged can also appear in the main image if a very strong reflector is encountered. Since ultrasound energy is focused at the center of the image field, the reflections from objects in front of or behind the imaging plane often appear faint (Fig. 5).

3. Shadowing occurs when a strong reflector has already transmitted most of the emitted sound waves back to the transducer, leaving minimal residual waves to reflect on deeper objects (Fig. 6).

4. Mirror imaging appears as a display of two images, one real and one artifact, due to the sound beam interacting with a strong reflector. In this situation, the sound waves are reflected by a structure as a mirror to another tissue interface. This gives the impression of a second structure beyond the first structure. The artifact is always deeper than the true anatomy and the distance between the mirror and the real anatomy on the proximal side and the artifact on the distal side are equal (Fig. 6).

5. Beam width artifacts occur when a poorly focused ultrasound beam is wider than the reflector being imaged. As the echogenicity of the reflector will be averaged with the adjacent normal tissue, subtle solid lesions might disappear from the image or cystic lesion may appear to be solid (Fig. 7).

### Optimizing images in neonatal echocardiography

The small size of the neonatal heart and its rapid rate of contraction make high spatial and temporal resolutions essential. Fortunately, the lack of need for deep tissue penetration allows use of high-frequency probes and high frame rates. Spatial and temporal resolutions are competing entities: obtaining high-resolution images takes longer, therefore decreasing temporal resolution. Key steps of image optimization in neonates include:

Use the highest transducer frequency available that provides adequate penetration, generally 8–12 MHz.

Increase temporal resolution by narrowing the sector width, decreasing the image depth, using zoom and using a single focus point.

Optimize focus point and image depth for each view and region of interest.

Use fundamental imaging rather than harmonic imaging, as the latter provides poorer axial resolution.

Adjust image gain to improve image contrast, but remember that this does not change signal-to-noise ratio.

Reduce the ambient light on the NICU to prevent the need to increase 2D gain. A high gain will reduce the contrast between the myocardial wall and chamber and reduce the quality of the image.

### Principles of Doppler ultrasound

**Fig. 8:**
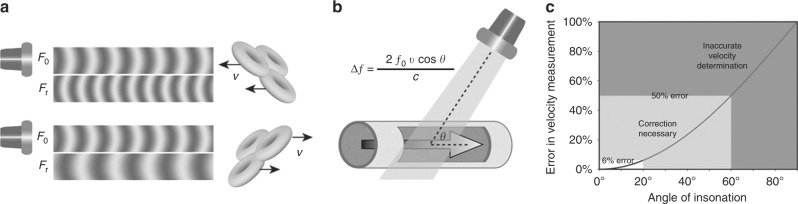
Doppler shift and angle of insonation (see text for details). Adapted from Rovner A. The principle of ultrasound—ECHOpedia 2017. Available from: http://www.echopedia.org/wiki/The_principle_of_ultrasound. *f*_0_ transmitted frequency, *f*_R_ received frequency; ∆*f* Doppler shift, *ν* blood velocity, cos Ø cosine of insonation angle, *c* ultrasound velocity in blood

The change in pitch of sound when a vehicle with a siren passes us in the street is a familiar example of the Doppler phenomenon. When traveling towards us the pitch of the sound is artificially increased as the vehicle has traveled closer to us with each sound emission, so the sound appears to have a higher frequency. Conversely, when traveling away from us the frequency appears lower. In ultrasound, the concepts are similar as the same shift in frequency occurs as sound is reflected off a moving object. The extent of this Doppler shift (the difference between the emitted and the received ultrasound frequencies) depends primarily on the velocity of the moving tissue and the angle of insonation between the ultrasound beam and the direction of movement (Fig. 8). Doppler applications can be used to quantify velocity of blood flow and myocardial tissue motion.

The angle of insonation of the ultrasound beam has a great impact on the extent of Doppler shift, such that minimization of the angle of insonation is a key step in all approaches to Doppler measurement. In practice, an angle of insonation of <20° is considered acceptable since this produces only a 6% reduction in velocity estimation (Fig. 8). If necessary correction can be made for remaining angle of insonation, but at high angles this process becomes more inaccurate. If the direction of movement is orthogonal to the imaging plane no Doppler shift is produced. While higher frequency probes provide optimal spatial resolution, lower frequencies may be required to provide adequate Doppler information, especially at higher flow velocities.

#### Continuous-wave Doppler and pulsed-wave Doppler

**Fig. 9:**
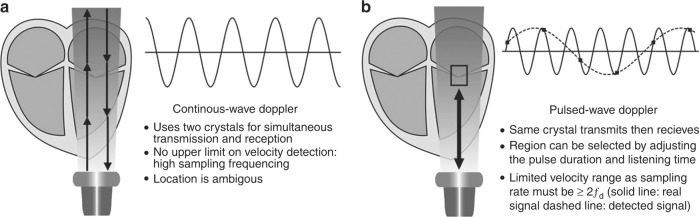
Continuous-wave (CW) and pulsed-wave (PW) Doppler. Signal sampling and aliasing (see text for details). Adapted from Rovner A. The principle of ultrasound— ECHOpedia 2017. Available from: http://www.echopedia.org/wiki/The_principle_of_ultrasound

In continuous-wave (CW) Doppler, two separate crystals simultaneously emit and receive signal. Continuous signal transmission can detect a wide range of velocities anywhere in the line of the ultrasound beam. Unlike pulsed-wave (PW) Doppler (below), CW Doppler has no upper limit for velocity detection. PW Doppler emits a single pulse, before pausing to detect received signals. By selecting a timeframe for receiving the data, one can measure exclusively velocities from a spatial range of interest. A wider range of interest (“sample gate”) increases signal but reduces spatial resolution. To measure a frequency shift, the sampling rate must be twice as high as the given frequency shift (Fig. 9). Therefore, at any given sampling rate there is a highest resolvable frequency (i.e., maximal velocity which can be detected), which is called the Nyquist limit. Above this Nyquist limit signal “aliases” to show an apparent opposite direction of motion (Fig. 9). Decreasing the imaging depth to increase the sampling frequency or decreasing the frequency of the ultrasound beam can be useful to overcome aliasing. A “wall thump filter” is applied to PW imaging to remove low-velocity, high-amplitude noises arising from the myocardium, but this setting must be adjusted when low-velocity flow is being sought (e.g., when looking for diastolic flow reversal in the descending aorta^20^ or venous flow).

#### Color flow imaging

Color Doppler is a technique for visualizing the velocity of blood within an image plane, such that local blood flow velocities are superimposed onto the corresponding B mode image. Velocities moving towards and away from the transducer are color-coded as red and blue, respectively. High variance of velocity (turbulent flow) is encoded by adding yellow or green to the pixels, whereas aliasing at the Nyquist limit is represented by color reversal. For computing a color flow map PW Doppler technique is employed. Given the complexity of the calculation and the high sampling requirement, the temporal resolution of color flow imaging is typically poor. But the technique remains extremely useful for detecting shunts and blood flow in regions of interest. Using the narrowest possible sector and minimal depth helps increase frame rate. Choice of gain settings is the key—accepted best practice being to increase the gain until background noise appears outside the vessels, then reducing it back until the noise is suppressed. However, this approach still leaves significant variability in gain settings which may produce clinically important variability in measurements, for example, in assessment of PDA diameter.^21^ For low-velocity signals it is important to reduce the velocity scale to enable proper visualization. It is advised to apply the following sequence within each image: 2D, color Doppler, PW Doppler, and CW Doppler if needed.

#### Tissue Doppler imaging

The physics of tissue Doppler imaging will be discussed in a separate review article in this series (see Nestaas et al., this issue).

### Bernoulli equation and flow volume calculation

A simplified version of the Bernoulli equation can be employed to estimate a pressure gradient between two points in the circulation, where pressure gradient (in mmHg) = 4*v*^2^, where *v* is the maximal velocity measured in m/s. The main utility of this technique in the neonate is in estimating right ventricular systolic pressure from the velocity of a tricuspid regurgitant jet (see de Boode et al., this issue).

Flow volumes are calculated by defining the cross-sectional area of the vessel of interest and measuring the velocity of flow by Doppler method. In the case of pulsatile flow pattern, the velocity time integral of the corresponding PW Doppler waveform is the area under a velocity time curve and is equivalent to the stroke distance. Multiplying this by the cross-sectional area of the vessel gives an estimate of stroke volume (SV). In neonates estimates of flow volume are subject to significant variability, particularly from estimation of vessel diameters, which are then squared to estimate area (and any associated errors are also squared). Cardiac output is then the product of SV and heart rate. Accuracy of these measurements is dependent on the quality of the 2D imaging, angle of insonation, and beat-to-beat variability. Errors are minimized by optimization of image quality and averaging multiple measurements.

## Patient safety during echocardiography

Minimal handling is one of the central tenets of neonatal care.^22^ While some studies have demonstrated clinicians’ ability to perform echocardiography without producing significant cardiorespiratory or thermal instability,^23,24^ lack of attention to infant status may have clinical consequences. Examinations should be targeted to key clinical questions where possible, while ensuring normal structural anatomy has been effectively confirmed.

1. Keep the baby warm by using warm gel. Single sachets can be placed in the isolette to warm and minimize infection control risks. Only one door on the incubator should be opened to avoid drafts. The sonographer should avoid getting gel on the infants’ temperature sensor, as the servo-controlled temperature mode will be disrupted. It is important to keep the baby swaddled if possible. This may also keep the baby more settled, allowing you to obtain images more quickly and thereby finish the scan sooner.

2. Keep the baby stable by using minimal pressure on the skin. Try to “float” the ultrasound probe above the skin. It is important for the sonographer to be especially sensitive in the subcostal views where too much pressure may cause the infant to vomit. One should ask the bedside nurse to monitor the child’s stability and provide timely feedback if the scan is impacting oxygenation—it can be hard to monitor this effectively while also performing the scan. Pause or discontinue scanning if the baby is unstable. The bedside nurse can also assist with repositioning the baby to aid image acquisition if necessary.

3. Keep the baby safe from infection by wiping down all usable surfaces of the ultrasound machine before and after every scan. Appropriate antimicrobial agents will vary with individual hospital policies and ultrasound vendors; specific agents are generally required for the ultrasound probe to avoid damaging the delicate head. We recommend auditing a regular clean of all surfaces on the ultrasound machine with a signed sheet attached to the machine. Single use gel sachets prevent avoidable infection control risks from an often-handled and frequently crusty ultrasound gel bottle.

4. Protect the baby’s skin by using the child’s existing electrocardiography (ECG) leads rather than applying a new set to provide an ECG trace for the scan. Most ultrasound vendors will provide you with a “slave” cable to allow the ultrasound machine to display the ECG trace from the infant’s cot-side monitor or an adapter to connect the child’s existing ECG leads to the ultrasound machine.

## Routine echocardiographic views

As previously discussed the first echocardiogram in a newborn should include a comprehensive structural assessment to confirm normal structural anatomy, even when the suspicion of congenital heart disease is low. While it is unrealistic for neonatologists to achieve the level of expertise of pediatric cardiologists in diagnosing structural congenital heart disease, we believe neonatologists performing echocardiograms should be capable of distinguishing normal and abnormal anatomy.

This is best achieved by following a structured approach to echocardiography, with the same standard views acquired in the same sequence at each scan. One should always perform all routine views, preferably in a routine sequence, unless this sequence is in conflict with the comfort of the newborn. A suggested sequence of structural views and “checklist” of normal anatomical features from each view is shown in Table 1.

**Table 1:** Suggested sequence of views and checklist of normal anatomical features and hemodynamic measurements from each view

	View	Structure	Function
1.	Subcostal situs view	•Normal abdominal situs •Pulsatile descending aorta •IVC drains to right atrium	•IVC filling and respiratory variation •(Descending aortic diastolic flow)
2.	Subcostal atrial and four-chamber views	•Intact intra-atrial septum (or patent foramen ovale) •Intact intraventricular septum •SVC drains into right atrium, pulmonary veins into left atrium •Sweep anterior to ascending aorta and pulmonary artery	•Filling of all cardiac chambers •Direction of PFO shunt •(Tricuspid regurgitant jet velocity) •(Pulmonary stroke distance)
3.	Apical four-chamber view	•Both ventricles reaching the apex of the heart •Opening mitral and tricuspid valves •Establish atrioventricular concordance—tricuspid valve positioned closer to the apex of the heart •Intact intraventricular septum •Rotate to “five-chamber view” to identify normal aortic valve from the left ventricle •Demonstrate pulmonary artery from the right ventricle crossing over aorta, excluding transposition •Pulmonary veins draining to left atrium	•Mitral inflow pattern •Tricuspid inflow pattern •Tricuspid regurgitant jet velocity •Aortic stroke distance •Pulmonary vein flow •Ejection fraction by area length •Consider tissue Doppler and speckle tracking
4.	Parasternal long axis view	•Normal motion of mitral and aortic valves •Intact intraventricular septum •Identify normal tricuspid valve •Identify normal pulmonary valve	•Aortic valve annulus •Pulmonary valve annulus •Pulmonary stroke distance •M mode for fractional shortening •M mode for LA:Ao ratio
5.	•Parasternal short axis view	•Identify normal (tricuspid) aortic valve •Intact intraventricular septum •Identify normal pulmonary valve •Identify bifurcation of main pulmonary artery into confluent right and left branches •Confirm drainage of pulmonary veins into left atrium (High parasternal “crab” view)	•Septum morphology (LV flattening) •(Tricuspid regurgitant jet velocity) •(M mode for fractional shortening) •(Pulmonary stroke distance) •Consider tissue Doppler and speckle tracking
6.	Ductal view	•Check ductal patency and direction of flow	•PDA diameter, flow direction and velocity •Descending aortic diastolic flow
7.	Arch view	•Normal arch (exclude interrupted arch and coarctation) including Doppler flow profile	

Measures in brackets may be obtained from these views if optimal alternate views are not available.

### Transverse subcostal view

**Fig. 10:**
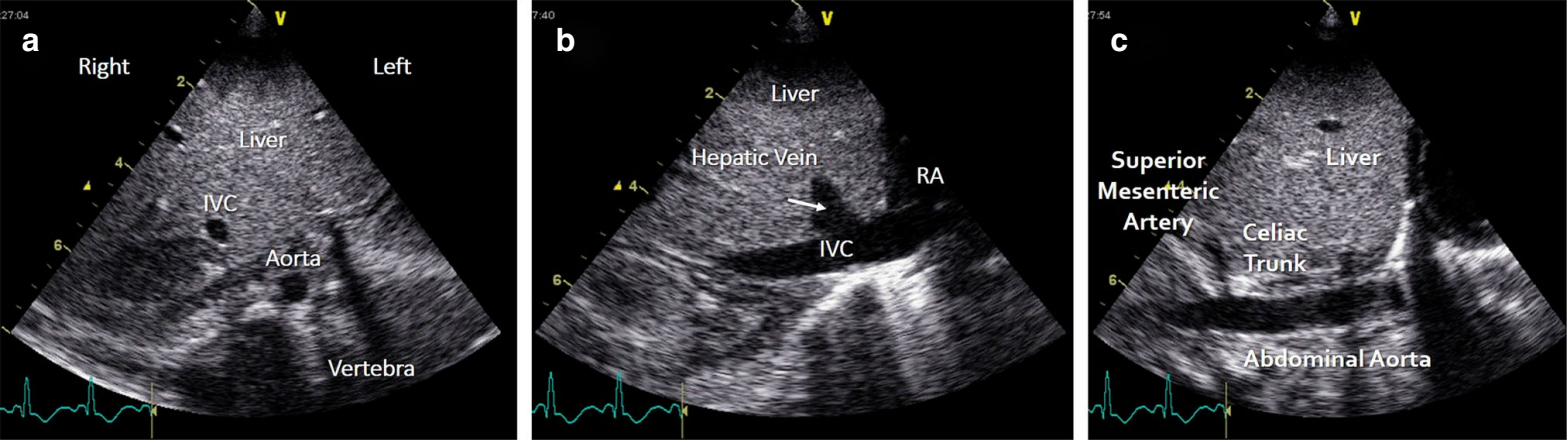
Transverse subcostal view (**a**), right parasagittal subcostal view (**b**), and left parasagittal subcostal view (**c**). IVC inferior vena cava, RA right atrium

“The ultrasound probe is placed under the xiphisternum, with the side marker on the probe pointing directly to the patient’s left. Ensure that the screen is set up such that the side marker (e.g., the yellow “V” in this image) is positioned to the right of the apex of the viewing triangle. From this view, much of the image is liver, but towards the bottom of the screen a circular pulsatile descending aorta, and an oval inferior vena cava are visible. The aorta should be to the subject’s left, that is, on the right of the screen.^25^” Normal situs of the aorta and IVC essentially confirms normal situs of the atria. Rotating the probe by 90° anticlockwise (pointing the side marker to the head will reveal the hepatic veins and IVC draining into the right atrium when the beam angled to the right of the infant and the abdominal aorta then the beam is angled to the left (Fig. 10).

### Subcostal atrial and four-chamber view

**“**With the ultrasound probe under the xiphisternum tilt to look towards the subject’s head, roughly at a 45° angle to the horizontal. The side marker on the probe should still be pointing to the subject’s left. The neonatal and pediatric cardiology consensus is to invert the screen vertically so that the apex of the imaging triangle is now at the bottom of the screen (see below) to obtain anatomically correct views. Adult echocardiographers may have the image inverted vertically, but apex down is a more intuitive view anatomically. This view contains key information, and is particularly valuable where other viewing windows are poor (e.g., in pneumothorax/chronic lung disease) as it essentially uses the liver as a window.^25^”

**Fig. 11:**
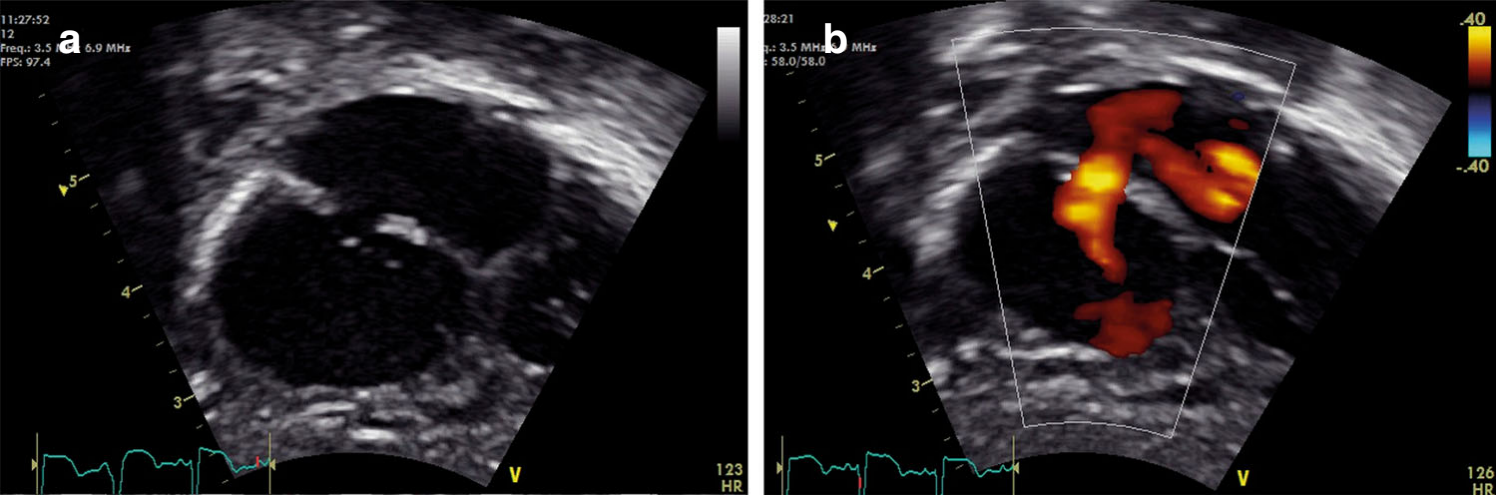
Subcostal atrial view 2D (**a**) and color Doppler showing left-to-right interatrial shunting (**b**)

“Look first for an intact intra-atrial septum using 2D imaging. Sweep anteriorly and posteriorly to visualize the entire intra-atrial septum. Use color Doppler to look for flow across the intra-atrial septum. A patent foramen ovale (PFO) is physiological in the newborn. Generally, flow through a PFO is predominantly left-to-right, and therefore will appear red on color Doppler as it flows towards the probe from the subcostal view (Fig. 11). Pure right to left interatrial shunt is congenital heart disease until proven otherwise.”

**Fig. 12:**
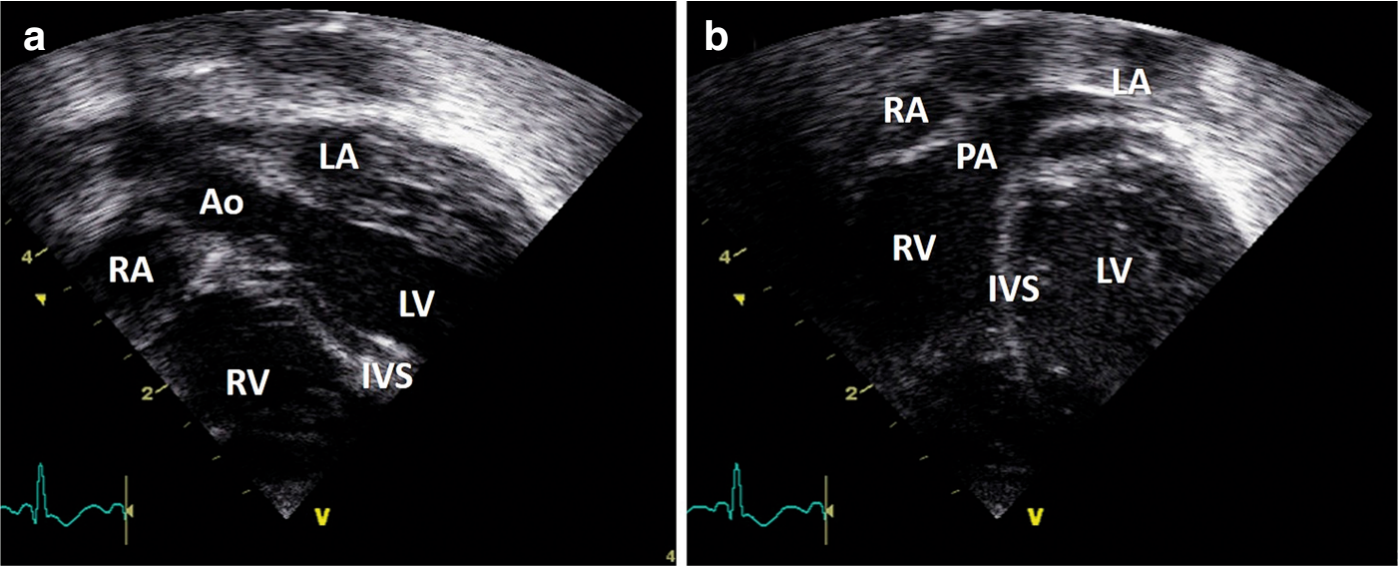
Subcostal view showing crossing great arteries; aorta (**a**) and pulmonary artery (**b**). IVS inter-ventricular septum, LV left ventricle, PA pulmonary artery, RV right ventricle, RA right atrium, LA left atrium

“From the atria sweep anteriorly to interrogate the membranous portion of the ventricular septum. Again, color Doppler helps exclude a defect (VSD). This is worth doing at this stage as from the apical four-chamber view the thinness of the membranous septum means that it may not reflect sound waves, and therefore mimic a VSD. Anterior angulation of the ultrasound beam in the subcostal view will reveal the superior vena cava, then almost immediately the aorta and finally the main pulmonary artery. To exclude transposition of the great arteries the vessels should be clearly seen to cross at this point (Fig. 12). ”

### Apical four-chamber view

**“**Place the echo probe on the apex beat. This can be difficult to palpate in small infants, so it is often best to start just below the left nipple. The side marker on the probe should still be pointing to the subject’s left. Again, by convention in neonatal and pediatric cardiology this view is produced with the apex at the bottom of the screen.^25^

**Fig. 13:**
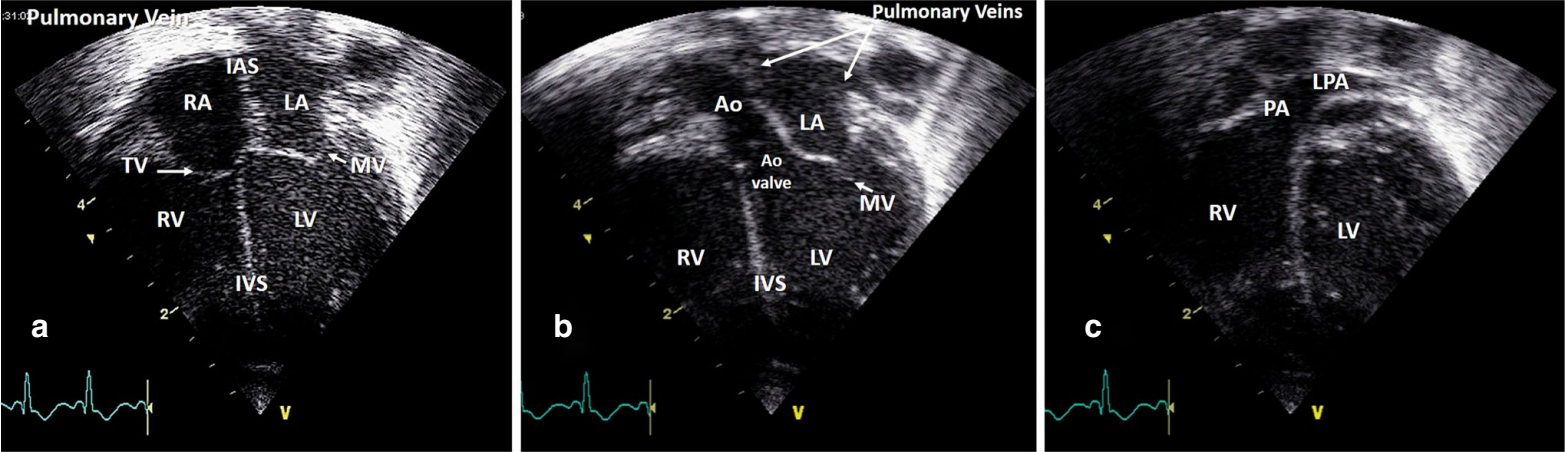
Apical four-chamber view (**a**), LV outflow tract (**b**), and RV outflow tract (**c**). IAS interatrial septum, IVS inter-ventricular septum, Ao aorta, LA left atrium, LV left ventricle, MV mitral valve, RA right atrium, RV right ventricle, TV tricuspid valve, LPA left pulmonary artery

Capture all four chambers of the heart in a single image. The heart should look like an egg standing on its end, with the ventricular septum vertical, and in the middle of the screen” (Fig. 13).

Establish the symmetry of the chambers and if chambers are disproportionately asymmetrical (one side dilated or hypoplastic) it often suggests significant abnormality. “The tricuspid valve should be placed more towards the apex of the heart than the mitral valve, referred to as ‘normal offsetting’. This is a subtle but important sign, as it essentially rules out atrioventricular septal defects. Use color Doppler to demonstrate normal flow through the mitral and tricuspid valves, and to exclude flow across the ventricular septum (indicating a ventricular septal defect (VSD)). Detection of a VSD is important not because it is generally hemodynamically significant in the newborn, but because it may indicate additional structural defects, for example, coarctation of the aorta. A key anatomical defect to rule out is total anomalous pulmonary venous connection (TAPVC), especially in children with PPHN.^25^” Moreover, this can be extremely difficult in the setting of severe PPHN, where volume of pulmonary blood flow is decreased, and the pulmonary venous return may be difficult to visualize. Color Doppler is often helpful and increasing the color Doppler gain and decreasing the color Doppler velocity range scale may aid in visualization. A clear flow of blood should be visible from the veins directly into the left atrium and then into the left ventricle. If in any doubt a pediatric cardiology opinion should be sought since TAPVC is the key differential diagnosis for PPHN, and surgical intervention rather than prostaglandin infusion may be required.

Lastly from the apical view, sweep anteriorly to image the great arteries. For the aorta, this is often referred to as the “five-chamber view.” Once again color Doppler is useful to delineate the vessels. Now, since blood is traveling away from the Doppler probe, the blood in the aorta and pulmonary will appear blue, and again the vessels should clearly be seen to cross each other. The pulmonary artery is anterior in position and bifurcates soon after its origin (Fig. 13).

### Parasternal long axis view

**Fig. 14:**
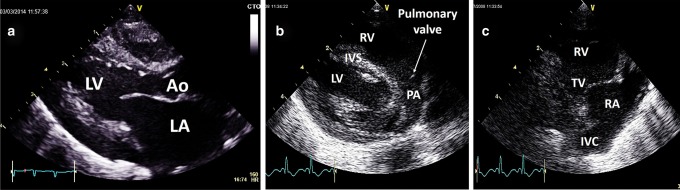
Parasternal long axis view of the LV (**a**), pulmonary artery (**b**), and tricuspid valve (**c**). Ao aorta, LV left ventricle, LA left atrium, IVS inter-ventricular septum, RV right ventricle, TV tricuspid valve, IVC inferior vena cava

Place the echo probe on the lower left sternal edge, around the fourth intercostal space. The side marker on the probe should now be pointing toward the subject’s right shoulder. By convention the apex of the heart is seen towards the left of the screen, the base of the heart to the right. A standard image should allow you to clearly see normal motion of the mitral and aortic valve leaflets (Fig. 14).

If in doubt about the valve motion, as with any view, use the high-resolution zoom feature to focus on the area of interest, and/or freeze the image and scroll back through preceding frames to confirm normal valve motion. This view is again helpful in establishing crossing of the great vessels, with the pulmonary artery lying anterior and to the left of aorta.

The long axis view is another important view to interrogate the entire inter-ventricular septum for a VSD. A membranous VSD sitting just below the aortic valve, and with an over-riding aorta suggests a diagnosis of tetralogy of Fallot. Since ultrasound produces only a 2D image of what is a 3D structure, use sweeps from side to side to ensure that all of the heart is interrogated. Sweeping to look towards the subject’s left shoulder visualizes the pulmonary valve; sweeping towards the subject’s right hip visualizes the tricuspid valve (Fig. 14). The valve leaflets should virtually disappear during systole, as they are so thin. Any thickening of the leaflets suggests valvar stenosis. Use color Doppler to interrogate flow across the valve.

### Parasternal short axis view

**Fig. 15:**
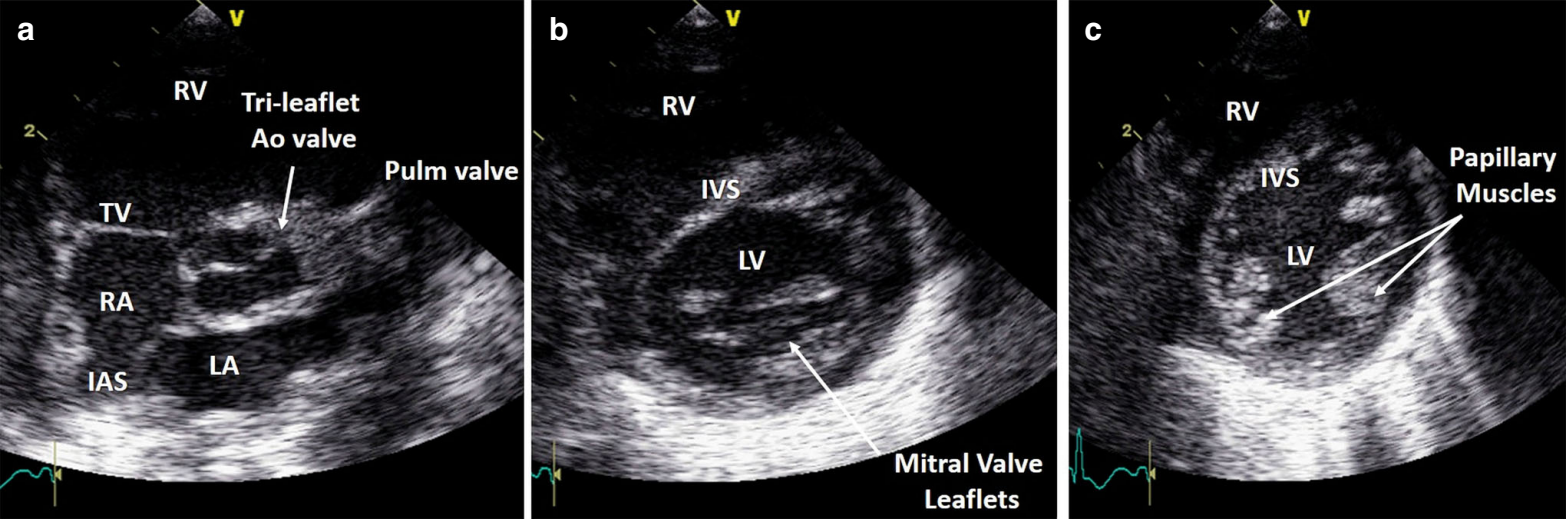
Parasternal short axis view at the level of the aorta (**a**), mitral valve (**b**), and papillary muscles (**c**). Ao aorta, LV left ventricle, LA left atrium, IVS inter-ventricular septum, IAS interatrial septum, RV right ventricle, TV tricuspid valve, IVC inferior vena cava

Echo probe placement is the same as for long axis imaging but the probe is rotated 90° clockwise, so that the side marker is pointing toward the subject’s left shoulder. At the initial view, a healthy tricuspid aortic valve is visible, sometimes called the (inverted) “Mercedes Benz” sign (Fig. 15). The pulmonary valve is anterior and to the left of the aortic valve (which appears at the center of the heart). Visualize the main pulmonary artery and the branch pulmonary arteries, and use color flow mapping to demonstrate confluent branch pulmonary arteries. By tilting to look towards the subject’s left hip a sweep of images through the left and right ventricles and the inter-ventricular septum from the base to the apex of the heart is obtained (Fig. 15).

### Ductal view

**Fig. 16:**
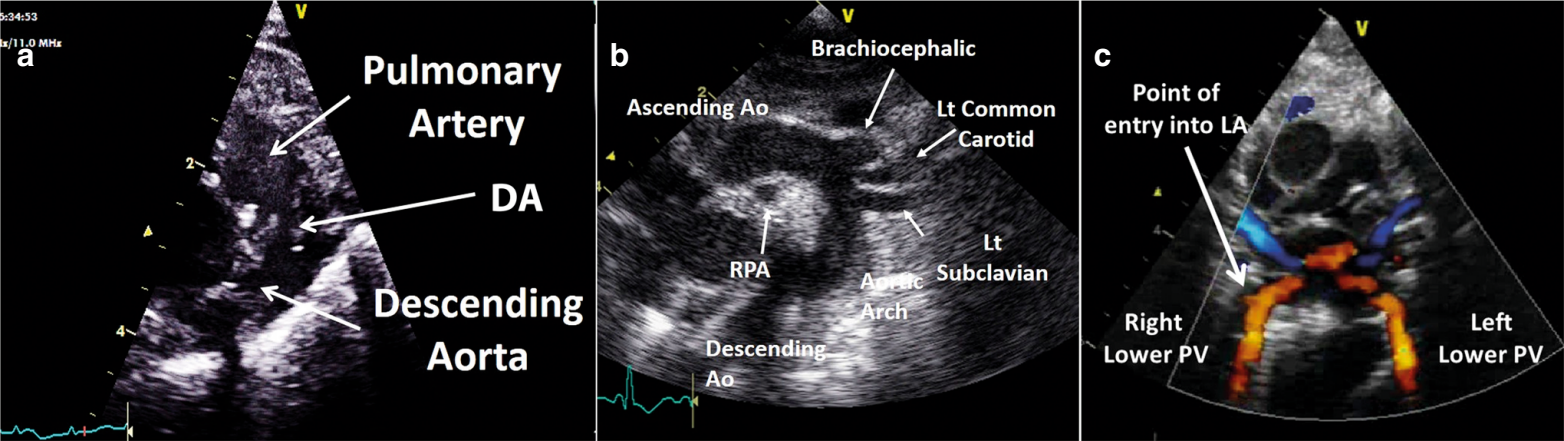
Ductal view (**a**), arch view (**b**), and (**c**). Ao aorta, DA ductus arteriosus, LA left atrium, Lt left, PV pulmonary vein, RPA right pulmonary artery

Use a high sagittal view with the side marker now pointing toward the subject’s chin. The proximal pulmonary trunk and distal aortic arch should be visible. A large PDA should be visible in B mode imaging, and color Doppler is helpful in identifying a PDA with left-to-right shunt (Fig. 16). However, note that in the presence of pure right to left shunt a PDA will appear as a blue structure in color Doppler, and can be difficult to differentiate from the right and left pulmonary arteries. With any component of left-to-right shunt, a patent duct should be clearly visible if a sweep from the pulmonary trunk to the descending aorta is obtained. Assessment of the hemodynamic significance of a PDA is discussed in a separate article (see van Laere et al., this issue).

### Arch view

**“**With the probe remaining high on the chest as for the ductal view, initially rotate around 45° clockwise. The marker should now be pointing towards the patient’s left shoulder. This is one of the hardest views to acquire, and often requires a repositioning of the probe to the right or left of the sternum.^25^” Having the patient’s head extended, for example, by putting a roll under the neck, often helps. To see the entire arch, the probe should be on the right side of the sternum rotated clockwise and the beam angled towards the apex until the arch appears. To interrogate further down the descending aorta (can be important to exclude coarctation), the transducer should be on the left side of the sternum in a true sagittal plane and tilted inferiorly. You should be able to see the full length of the arch (round to the left subclavian artery) on a single view (Fig. 16).

It is important to see the full length of the arch as most coarctations occur at or below the level of the left subclavian artery. Color Doppler can be very helpful to look for turbulent flow at the point of a potential coarctation. A high velocity diastolic run-off pattern will also suggest the presence of coarctation. Flow should look laminar and generally <1.5 m/s, though a degree of acceleration is common. From the same view follow the descending aorta distally towards the diaphragm and place a PW Doppler gate across the descending aorta below the level of the duct to look for diastolic flow reversal—a key marker of high volume PDA shunt.

### Suprasternal view

With the side marker pointed towards the left, the probe is positioned in the suprasternal notch. Angling the probe towards the anterior chest wall will show the pulmonary veins connecting to the left atrium (Fig. 16). This so-called “crab view” is rather difficult to obtain.

## Summary

NPE has an increasing role in hemodynamic assessments in the neonatal intensive care unit. A well-defined list of indications for scanning gives the neonatologist with echocardiographic skills a clear scope of practice. An understanding of the physics of ultrasound allows imaging optimization (essential to minimize errors in quantitative measures) and avoidance of confusion from artifacts. Common sense steps keep the process safe for the infant. Following a set sequence of views with definite anatomic features to be confirmed at each view allows the neonatologist to exclude significant structural congenital heart defects which present in the neonatal period, and/or to acquire the necessary images for a pediatric cardiologist to do so when reviewing the scan. Interpretation of all hemodynamic measurements of course requires integration with the clinical picture. Regular follow-up assessments may provide additional insight regarding diagnosis and effect of intervention.

## Appendix

**European Special Interest Group “Neonatologist Performed Echocardiography” (NPE), endorsed by the European Society for Paediatric Research (ESPR) and European Society for Neonatology (ESN)**

**de Boode W. P.** (chairman), Department of Neonatology, Radboud University Medical Center, Radboud Institute for Health Sciences, Amalia Children’s Hospital, Nijmegen, The Netherlands (willem.deboode@radboudumc.nl)

**Austin T.**, Department of Neonatology, Rosie Hospital, Cambridge University Hospitals NHS Foundation Trust, Cambridge, United Kingdom (topun.austin@addenbrookes.nhs.uk)

**Bohlin K.**, Department of Neonatology, Karolinska University Hospital, Karolinska Institutet, Stockholm, Sweden (kajsa.bohlin@ki.se)

**Bravo M. C.**, Department of Neonatology, La Paz University Hospital, Madrid, Spain (mcarmen.bravo@salud.madrid.org)

**Breatnach C. R.**, Department of Neonatology, The Rotunda Hospital, Dublin, Ireland (colm.breatnach@gmail.com)

**Breindahl M.**, Karolinska University Hospital, Karolinska Institutet, Stockholm, Sweden (morten.breindahl@sll.se)

**Dempsey E.**, INFANT Centre, Cork University Maternity Hospital, University College Cork, Ireland (g.dempsey@ucc.ie)

**El-Khuffash A.**, Department of Neonatology, The Rotunda Hospital, Dublin, Ireland & Department of Pediatrics, The Royal College of Surgeons in Ireland, Dublin, Ireland (afifelkhuffash@rcsi.ie)

**Groves A. M.**, Division of Newborn Medicine, Mount Sinai Kravis Children’s Hospital, New York, NY, USA. (alan.groves@mssm.edu)

**Gupta S.**, University Hospital of North Tees, Durham University, Stockton-on-Tees, United Kingdom (samir.gupta@nth.nhs.uk)

**Horsberg Eriksen B.**, Department of Pediatrics, Møre and Romsdal Hospital Trust, Ålesund, Norway (beate.eriksen@me.com)

**Levy P. T.**, Department of Pediatrics, Washington University School of Medicine, Saint Louis, MO, USA & Department of Pediatrics, Goryeb Children’s Hospital, Morristown, NJ, USA (Levy_P@kids.wustl.edu)

**McNamara P. J.**, Departments of Pediatrics and Physiology, University of Toronto, Toronto, Canada (patrick.mcnamara@sickkids.ca)

**Molnar Z.**, John Radcliffe Hospital, Oxford, United Kingdom (zoltan.Molnar@ouh.nhs.uk)

**Nestaas E.**, Institute of Clinical Medicine, Faculty of Medicine, University of Oslo, Norway; Department of Cardiology and Center for Cardiological Innovation, Oslo University Hospital, Rikshospitalet, Oslo, Norway & Department of Paediatrics, Vestfold Hospital Trust, Tønsberg, Norway (nestaas@hotmail.com)

**Rogerson S. R.**, The Royal Women’s Hospital, Parkville, Victoria, Australia (sheryle.Rogerson@thewomens.org.au)

**Roehr C. C.**, Department of Paediatrics, University of Oxford, John Radcliffe Hospital, Oxford, United Kingdom (charles.roehr@paediatrics.ox.ac.uk)

**Savoia M.**, Azienda Ospedaliero-Universitaria S Maria della Misericordia, Udine, Italy (marilena.savoia@gmail.com)

**Schubert U.**, Department of Clinical Science, Intervention and Technology, Karolinska Institutet, Stockholm, Sweden (ulfschubert@gmx.de)

**Schwarz C. E.**, Department of Neonatology, University Children’s Hospital of Tübingen, Tübingen, Germany (c.schwarz@med.uni-tuebingen.de)

**Sehgal A.**, Department of Pediatrics, Monash University, Melbourne, Australia (arvind.sehgal@monash.edu)

**Singh Y.**, Addenbrooke’s Hospital, Cambridge University Hospitals NHS Foundation Trust, Cambridge, United Kingdom (yogen.Singh@nhs.net)

**Slieker M. G.**, Department of Paediatric Cardiology, Radboudumc Amalia Children’s Hospital, Nijmegen, The Netherlands (Martijn.Slieker@radboudumc.nl)

**Tissot C.**, Department of Pediatrics, Clinique des Grangettes, Chêne Bougeries, Switzerland (cecile.tissot@hotmail.com)

**van der Lee R.**, Department of Neonatology, Radboud University Medical Center, Radboud Institute for Health Sciences, Amalia Children’s Hospital, Nijmegen, The Netherlands (Robin.vanderLee@radboudumc.nl)

**van Laere D.**, Department of Pediatrics, Antwerp University Hospital UZA, Edegem, Belgium (david.VanLaere@uza.be)

**van Overmeire B.**, Department of Neonatology, University Hospital Brussels, Brussels, Belgium (bart.van.overmeire@erasme.ulb.ac.be)

**van Wyk L.**, Department of Paediatrics & Child Health, University of Stellenbosch, Cape Town, South Africa (lizelle@sun.ac.za)
